# Multidrug-Resistant Tuberculosis Outbreak in Gaming Centers, Singapore, 2012

**DOI:** 10.3201/eid2101.141159

**Published:** 2015-01

**Authors:** Cynthia B.E. Chee, Suay-Hong Gan, Rick T. Ong, Li-Hwei Sng, Christopher W. Wong, Jeffery Cutter, Min Gong, Hui-Maan Seah, Li Yang Hsu, Suhana Solhan, Peng-Lim Ooi, Eryu Xia, Jayne T. Lim, Chwee-Kim Koh, Soon-Kok Lim, Han-Kee Lim, Yee-Tang Wang

**Affiliations:** Tan Tock Seng Hospital, Singapore (C.B.E. Chee, S.-H. Gan, L.Y. Hsu, C.-K. Koh, H.-K. Lim, Y.-T. Wang);; Saw Swee Hock School of Public Health, Singapore (R.T. Ong, L.Y. Hsu);; Singapore General Hospital, Singapore (L.-H. Sng);; Genome Institute of Singapore, Singapore (C.W. Wong, M. Gong, H.-M. Seah);; Ministry of Health, Singapore (J. Cutter, S. Solhan, P.-L. Ooi, J.T. Lim;; Yong Loo Lin School of Medicine, Singapore (L.Y. Hsu);; NUS Graduate School for Integrative Science and Engineering, Singapore (E. Xia)

**Keywords:** multidrug-resistant tuberculosis, MDR TB, outbreak, whole-genome sequencing, cyber café, contact tracing, bacteria, Singapore, gaming centers, tuberculosis and other mycobacteria

**To the Editor:** Local area network (LAN) gaming centers (variant of cyber cafes) have proliferated over the past 2 decades. Patrons sometimes spend considerable time playing multiplayer computer games at these centers. We report a 2012 outbreak of multidrug-resistant tuberculosis (MDR TB) in Singapore, in which transmission occurred among 5 immunocompetent 19- to 28-year-old men within 2 LAN gaming centers. This report highlights LAN gaming centers as potential hotspots for TB transmission and notes challenges faced when conducting contact-tracing investigations in such settings.

The outbreak timeline is shown in the Technical Appendix Figure. Patients A–D had frequented LAN center 1, but 3 months before patient A received a TB diagnosis, the center closed, and they continued their gaming activities at LAN center 2. Patient E had only patronized LAN center 2. In February 2012, the initial case-patient, patient A, sought medical care for a cough of 4 months’ duration. Chest radiographs showed bilateral cavitary lesions, a sputum smear was positive for acid-fast bacilli, and a sputum culture grew *M. tuberculosis* with phenotypic resistance to rifampin, isoniazid, streptomycin, and ethionamide. Contact tracing for patient A was delayed because he defaulted on directly observed therapy after 5 days and eluded contact for 6 weeks.

Before his diagnosis, patient A had spent several hours daily at LAN center 1, where he participated in gaming and worked part time. Thirty contacts from center 1 were identified, but most failed to show up for screening until Ministry of Health public health officers intervened 4 months after patient A received his diagnosis. The attack rate was 40% among the 30 contacts. Two contacts (patients B and C) were sputum TB culture–positive and had bacteria with identical drug-resistance phenotypes; 12 contacts were positive for latent TB (QuantiFERON-TB Gold In-Tube test; Cellestis Ltd, Carnegie, VIC, Australia). Patients D and E (not initially identified as patient A contacts) sought care for pulmonary TB 8 and 10 months, respectively, after patient A’s diagnosis. Questioning revealed that they had patronized LAN center 2 during the same period as patients A–C. Culture results for patients D and E were positive for MDR TB, and bacteria had a drug-resistance phenotype identical to that for isolates from patients A–C.

Attempts to expand contact screening at LAN center 2 were met with resistance from the center’s management. Thus, a nationwide alert was issued by the Ministry of Health, and legal orders and health advisories were served to LAN center employees and patrons, yielding 44 additional contacts, of whom, only 1 had positive results by the QuantiFERON-TB Gold In-Tube test. Contacts with latent TB infection were not treated because evidence is inconclusive for the efficacy of preventive therapy; these contacts had follow-up medical visits every 6 months for 2 years.

Results of spoligotyping (Ocimum Biosolutions, Hyderabad, India) and 24-loci mycobacterial interspersed repetitive units (MIRU) typing and variable number tandem repeats (VNTR) analysis (MIRU-VNTR Typing kit; GenoScreen, Lille, France) were identical for all 5 isolates; the isolates were shown to belong to the Beijing lineage (*1*). Our MIRU-VNTR database contains data for 112 *M. tuberculosis* isolates, representing 87.5% of 128 MDR TB cases diagnosed in Singapore during 2008–2012. Of these isolates, only 1 was identical to isolates in this investigation. That isolate derived from a karaoke lounge hostess (patient K) who received a diagnosis of pulmonary TB after a positive sputum smear in 2008 but left Singapore shortly after the diagnosis and was lost to follow-up.

Because MIRU-VNTR lacks discriminatory power for Beijing lineage isolates (*2*), we performed whole-genome sequencing (HiSeq 2000; Illumina, San Diego, CA, USA) on the 6 identical isolates to determine epidemiologic links. Paired-end reads were mapped to the H37Rv reference genome (GenBank accession no. NC000962.3) by using the Burrows–Wheeler aligner (*3*). Bioinformatics analysis and single-nucleotide polymorphism identification were performed as described (*4*,*5*). Isolates from patients A–E were identical, and that from patient K differed by 3 single-nucleotide polymorphisms (Technical Appendix Figure). Questioning revealed that patient A had worked at the karaoke lounge when patient K was employed as a hostess, but he did not recall encountering her. None of the other patients had visited the lounge and did not recall contact with patient K.

The proliferation of LAN gaming centers exemplifies how modern technology and urbanization have spawned new patterns of behavior and foci of TB transmission. In Asian countries, such centers are usually enclosed, air-conditioned spaces where patrons sometimes spend up to several hours each day, putting them at high risk for TB infection if 1 person among them is infectious. Contact-tracing investigations are challenging in such situations because contacts are not easily identified and may be reluctant to appear for screening. Investigation is difficult even within legal frameworks, such as that provided by Singapore’s Infectious Diseases Act (*6*), which can be invoked to compel persons with an infectious disease and their contacts to submit to medical evaluation and treatment. Our investigation provides additional affirmation for the role of whole-genome sequencing in constructing a transmission chain, which in this outbreak enabled identification of the index patient.

Technical Appendix Timeline of a multidrug-resistant tuberculosis (MDR TB) outbreak associated with a local area network (LAN) gaming cafe in Singapore.
