# Supplementary material for: Multidrug-Resistant Tuberculosis Outbreak in Gaming Centers, Singapore, 2012
Source: Emerg Infect Dis. 2015 Jan;21(1):179–80. doi: 10.3201/eid2101.141159 (PMC4285258; doi:10.3201/eid2101.141159)
Supplement: Technical Appendix — Timeline of a multidrug-resistant tuberculosis (MDR TB) outbreak associated with a local area network (LAN) gaming cafe in Singapore. [file 14-1159-Techapp-s1.pdf]

Article DOI: http://dx.doi.org/10.3201/eid2101.141159

Multidrug-Resistant Tuberculosis Outbreak in Gaming Centers, Singapore, 2012

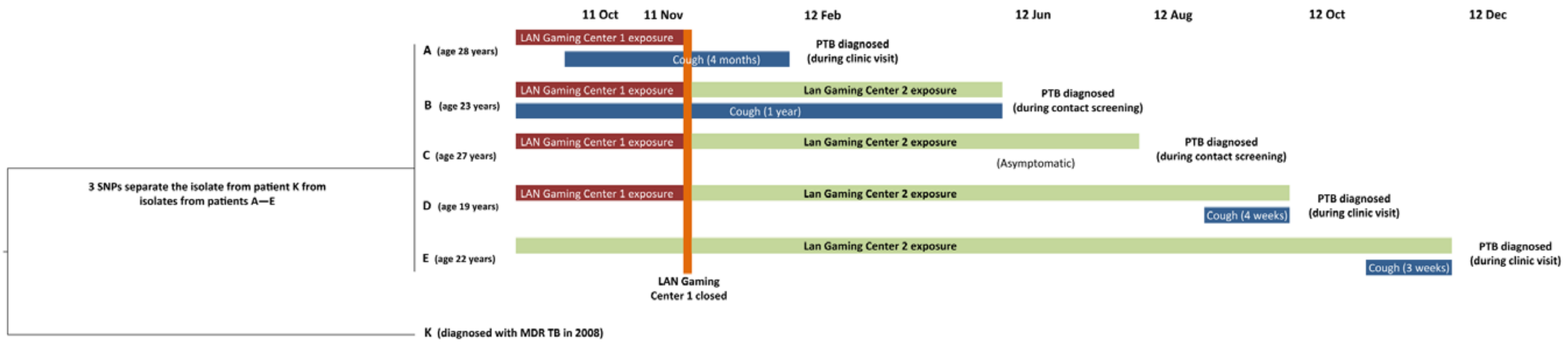

Technical Appendix Figure. Timeline of a multidrug-resistant tuberculosis (MDR TB) outbreak associated with a local area network (LAN) gaming cafe in Singapore. During February–December 2012, MDR TB infection was confirmed in 5 persons (case-patients A–E) who patronized the cafe. A minimum spanning tree based on single nucleotide polymorphism (SNP) analysis of whole-genome sequencing results of the MDR TB isolates is appended; the analysis includes the isolate from putative index case K, who received a diagnosis of pulmonary MDR TB in 2008 and who worked as a hostess at a karaoke lounge where patient A had also worked during the same period. PTB, pulmonary tuberculosis.
